# Current Infections of the Orofacial Region: Treatment, Diagnosis, and Epidemiology

**DOI:** 10.3390/life13020269

**Published:** 2023-01-18

**Authors:** Elahe Tahmasebi, Ali Keshvad, Mostafa Alam, Kamyar Abbasi, Saeide Rahimi, Farzad Nouri, Mohsen Yazdanian, Hamid Tebyaniyan, Artak Heboyan, Gustavo Vicentis Oliveira Fernandes

**Affiliations:** 1 Research Center for Prevention of Oral and Dental Diseases, Baqiyatallah University of Medical Sciences, Tehran 1435916471, Iran; 2 Department of Orthodontics, School of Dentistry, Tehran University of Medical Sciences, Tehran 1416634793, Iran; 3 Department of Oral and Maxillofacial Surgery, School of Dentistry, Shahid Beheshti University of Medical Sciences, Tehran 1516745811, Iran; 4 Department of Prosthodontics, School of Dentistry, Shahid Beheshti University of Medical Sciences, Tehran 1516745811, Iran; 5 Department of Pediatric Dentistry, School of Dentistry, Ahvaz Jundishapur University of Medical Sciences, Ahwaz 6135715794, Iran; 6 Department of Oral and Maxillofacial Surgery, School of Dentistry, Zahedan University of Medical Sciences, Zahedan 9816743463, Iran; 7 Department of Science and Research, Islimic Azade University, Tehran 1477893855, Iran; 8 Department of Prosthodontics, Faculty of Stomatology, Yerevan State Medical University after Mkhitar Heratsi, Str. Koryun 2, Yerevan 0025, Armenia; 9 Periodontics and Oral Medicine Department, University of Michigan School of Dentistry, Ann Arbor, MI 48109, USA

**Keywords:** oral fungal infections, oral bacterial infections, oral viral infections, orofacial infection, orofacial microbes

## Abstract

Undoubtedly, diagnosing and managing infections is one of the most challenging issues for orofacial clinicians. As a result of the diversity of symptoms, complicated behavior, and sometimes confusing nature of these conditions, it has become increasingly difficult to diagnose and treat them. It also highlights the need to gain a deeper insight into the orofacial microbiome as we try to improve our understanding of it. In addition to changes in patients’ lifestyles, such as changes in diet, smoking habits, sexual practices, immunosuppressive conditions, and occupational exposures, there have been changes in patients’ lifestyles that complicate the issue. Recent years have seen the development of new infection treatments due to the increased knowledge about the biology and physiology of infections. This review aimed to provide a comprehensive overview of the types of infections in the mouth, including the types that viruses, fungi, or bacteria may cause. It is important to note that we searched the published literature in the Scopus, Medline, Google Scholar, and Cochran databases from 2010 to 2021 using the following keywords: “Orofacial/Oral Infections,” “Viral/Fungal/Bacterial Infections”, “Oral Microbiota” And “Oral Microflora” without limiting our search to languages and study designs. According to the evidence, the most common infections in the clinic include herpes simplex virus, human papillomavirus, *Candida albicans*, *Aspergillus*, Actinomycosis, and *Streptococcus mutans*. The purpose of this study is to review the new findings on characteristics, epidemiology, risk factors, clinical manifestations, diagnosis, and new treatment for these types of infectious diseases.

## 1. Introduction

The natural flora of the mouth is very diverse. After the large intestine, it has the second highest complexity in terms of microorganisms, including more than 700 microorganisms [1]. The tongue is the primary source of microbes in saliva and the major oral site for microbial growth [2]. Some bacterial, viral, and fungal agents can affect the skin and mucous membranes in the orofacial area. These factors can cross natural barriers and cause lesions of varying degrees. Some of them can even be life-threatening. The local and systemic factors determining the microbiota and genetics, as well as the individual factors such as diet, teeth brushing habits, dentures, dental procedures, mouthwashes, medications, etc., can be involved in the occurrence of orofacial infections [1,3,4,5,6,7]. Oral infections can occur under certain conditions, such as poor oral hygiene, antibiotic consumption, trauma, and dry mouth, and can damage the oral cavity. These infections also may spread into the nearby tissues and turn into systemic infections. For example, chronic oral infections are known to be a risk factor for cardiac disease [2].

Each tissue has specific microbes that are different from adjacent tissues’ microbes. Still, these microbes can be displaced under certain conditions, such as the effects of cytotoxic drugs, oral cancer, or epithelial atrophy [8,9]. The oral flora is divided into two categories, static and transient, which regularly balance with the host and protect against pathogenic microorganisms. The static flora on oral cavity surfaces is known as biofilm that can improve or protect oral health against pathogens, increase the virulence of potentially harmful microorganisms, and reduce the effectiveness of antimicrobial agents [10,11,12]. Infections caused by bacteria in the mouth include caries and periodontitis [8]. Microorganisms can attack different parts of the mouth via different mechanisms. For example, keratinized epithelial surfaces, such as the palate, become excessively keratinized, and their contained opportunistic microorganisms may lead to infection. The buccal and labial mucosa, as non-keratinized epithelial surfaces, may be occupied by dead or apoptotic cells that may be attacked by bacteria [10,13,14,15]. Despite the progression of several local or generalized treatment methods, orofacial infections may still cause severe discomfort and pain. Therefore, the popularity of the clinical presentation of those infections is dominant to their diagnosis, clinical management, and appropriate referral. The following review includes the most recognized orofacial infective agents and their clinical characteristics.

## 2. Herpes Simplex Virus

Herpes simplex virus [16] is one of 200 species of the *Herpesviridae* family and belongs to the subfamily *Alphaherpesvirinae*, the genus *Simplexvirus*. Despite differences in host and morphology, this family is thought to be originated from a common ancestor with tailed bacteriophages [17]. These viruses are about 120 to 200 nanometers in diameter. Structurally, they include a circled DNA containing a unique long (UL) segment and a unique short (US) segment (opposite repeats) [17,18]. In addition, they have an icosahedral symmetric capsid, protein coating, and a lipid bilayer cell-like envelope. Genome transcription and replication and the formation of new capsids occur in the host cell’s nucleus [18]. These viruses’ short reproductive cycle helps them rapidly destroy the host cells [17]. During the infection procedure, the envelope releases the capsid into the host cell’s cytoplasm after fusion with its plasma membrane. HSV can attack nerve tissue and multiply there (neuro-virulence). Eighty-three percent identical nucleotides and fifty percent homology in the sequence display a strong association between the two types of herpes virus: HSV-1 and 2 [17].

**Figure 1:**
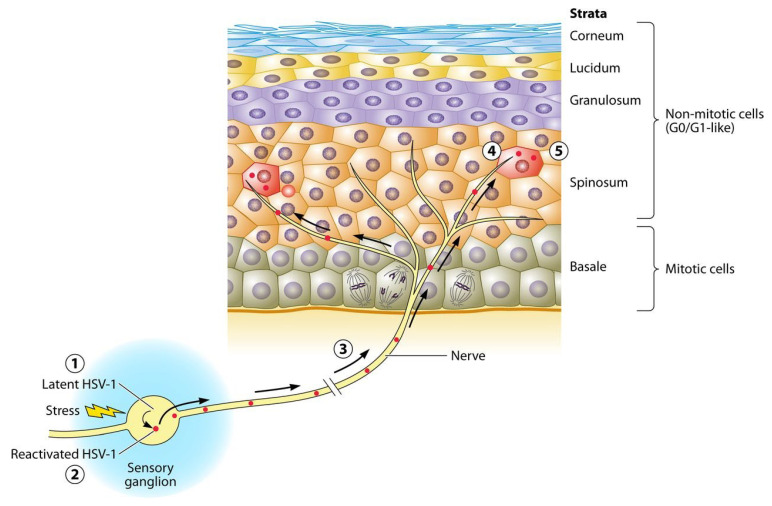
The latent infection of HSV-1 leads to permanent arrest in an G0/G1-like state in the sensory ganglia that will never reenter mitosis ①. During reactivation, virus genes are expressed in cells in the most permissive state ②. Then, reactivated virions travels in axons ③ to termini on the epidermis ④, and then transmitted to the spinosum and granulosum strata layers of the epidermis ⑤ [17].

HSV is also recognized for its latency ability in trigeminal and sacral ganglion nuclei of the ganglia structures [17,19]. HSV fuses to the axon termini to establish latency and moves retrogradely along the sensory fibers (Figure 1) [17]. Then, it generates latency-associated transcripts (LATs) that enhance axon regeneration, reduce viral gene expression, and prevent nerve cell apoptosis [20].

### 2.1. Epidemiology

HSV is most commonly transmitted through asymptomatic shedding from an infected person’s mouth or genitals. It is mainly transmitted through proximity to an infected person in immunocompromised and immunocompetent individuals [21]. Recurrent HSV infections occur in one-third of the world’s population. HSV-1 and 2 usually infect the mucocutaneous of the mouth and genitals, respectively. The incidence of HSV is 40–95% worldwide, depending on the socio-economic status of the studied population, with higher rates in lower socio-economic populations. Sixty percent of adults with HSV-2 have orolabial herpes [21]. Generally, the seroprevalence of HSV increases with age and shows some correlations to the country’s income. Sixty percent higher seroprevalence in people over the age of 40 than in those under 20 suggests an increased risk of exposure that should be observed by health beneficiaries [22].

### 2.2. Clinical Presentation

The disease has no clinical signs at the initial acquisition or during periods of reactivation. However, symptoms occur in the active periods of the disease, following asymptomatic periods, on a rotating basis [23]. Clinical symptoms of the first episode of the disease include headache, fever, muscle aches, and inflammation of the lymph nodes [23]. Then, classic lesions are created that include fluid-filled vesicles that become purulent and dry. Genital herpes includes small blisters resulting in benign ulcers, whereas, in cold sores, several small blisters are aggregated [23]. Other vital clinical signs of common gingivitis include bleeding gums with edema and friable erythematosus. In addition, sores on the buccal mucosa and gums may be yellow based on a red halo. These oral lesions are often associated with anorexia. Symptoms in young children can include drooling, halitosis, and dehydration [23]. Herpetic vitiligo is another manifestation, which is painful, erythematous, and swollen lesions often occur in the distal phalanx of the hand, caused by HSV-1 or HSV-2. This lesion mainly affects the fingers (thumb) and rarely the palm [23]. HSV-1 eczema herpeticum of Kaposi varicelliform eruption is caused by poor immunity due to atopic dermatitis, burns, or topical immunosuppressants. The disease includes vesicular skin lesions [24]. HSV eye keratitis and HSV encephalitis are other complications caused by this virus. Eye involvement can lead to sensitive retinal necrosis, conjunctivitis, blepharitis, or chorioretinitis. Reaching this viral infection to the brain parenchyma leads to extensive hemorrhagic necrosis and vascular occlusion. Encephalitis is the worst outcome that can occur in a healthy person following this infection [24,25]. Patients with good immunity have milder symptoms in the recurrence period than in the early symptoms. In general, patients with HSV-1 usually have lesions around the lips and mouth. Several studies have reported a local burning prodrome with roughness or itching before the vesicles erupt. The lymph nodes in the affected area can become sensitive and enlarged without systemic symptoms and fever. Studies have shown that HSV-1 and Epstein–Barr virus (EBV) is associated with invasive periodontal disease caused by bacteria [26].

### 2.3. Diagnosis

Because most people with HSV-2 do not have the classic clinical symptoms, genital herpes is more challenging to diagnose than oral herpes and can be confused with related diseases such as fungal infections, lichen planus, atopic dermatitis, and urinary tract diseases. Diagnosis is confirmed by laboratory tests, including polymerase chain reaction (PCR), skin biopsy, immunofluorescence, and virus culture polymerase chain reaction (PCR). Blood and urine cultures determine previous and ongoing infections, respectively. PCR is more sensitive and accurate for diagnosing HSV in mucosal surfaces than vesicular fluid culture. In patients with a history of genital ulcers lacking active lesions, serological diagnostic tests are conducted [27].

### 2.4. Treatment

Although antiviral drugs, including acyclovir, valaciclovir, famciclovir, and penciclovir, reduce the disease’s incidence, duration, and severity, no way exists to remove the virus from the body. Painkillers and topical anesthetic treatments can relieve pain/fever and itching, respectively; however, their efficiency has been controversial [28]. In addition, intravenous infusion of acyclovir is recommended for eczema herpeticum to be limited and prevented from spreading to the eye [25]. Seizure control, fluids management, and intracranial pressure control will improve results in HSV-based eye keratitis [24,25]. Suppressive therapy, such as corticosteroids, is commonly used to improve pain and tenderness at the onset of symptoms. Oral acyclovir has a positive therapeutic effect on primary and recurrent HSV genital infections [25]. Some studies have discussed the antiviral activity of some essential oils on the HSV spp. (even acyclovir-resistant strains) through different mechanisms, such as inhibiting cell attachment. These studies also imply that the synergistic activity of the extracts and, thus, essential oils minimize the development of resistant virus strains [29,30]. A recent development in treating HSV is presented in Table 1.

## 3. Human Papilloma Viruses

Papillomaviruses have a wide genetic diversity. Human papillomavirus (HPV) viruses use human cellular proteins to reproduce and survive [52]. The virus genome consists of open reading frames [53] and long control regions (LCR) to regulate the replication and transcription of primary genes [54]. The main reservoir of HPV is inflamed gums, salivary gland epithelium, and cryptal epithelium of tonsils, oral border, and oropharynx. The most clinically important genus of HPV is the alpha genus of human papillomavirus [52]. In high-risk HPV, placing the virus genome in the host genome breaks the virus genome at the E1 and E2 sites, and losing E2, in turn, causes E6 and E7 to lose control that, inhibits the regular function of p53 and pRb, respectively, and interfere with the cell cycle [55].

### 3.1. Epidemiology

Owing to effective screening strategies, the incidence of HPV-caused cervical cancer has decreased; however, the oropharyngeal squamous cell carcinoma (OPSCC) cases have kept rising. In addition, the prevalence of HPV DNA has increased by between 24–45 percent from 1990 to 2005 [56]. In a study in the US, oral HPV rates in men were around 60 percent higher than in women. Additionally, their study evidenced the HPV is the leading cause of oropharyngeal carcinoma (OPC) in men, whereas smoking is the predominant cause in women [57].

### 3.2. Clinical Presentation

HPV infection can be transmitted from the mother’s cervix (sexual and non-sexual fomite transmission) and produces clinical or subclinical lesions. Oral HPV lesions include a range of benign oral lesions, lichen planus, fibroma, hyperplastic, papillomatous, verrucous, and carcinoma lesions. Generally, flat, exophytic, or wart-like white lesions in the oral mucosa, exophytic, wart-like, or papillary proliferations can be considered clinical manifestations of HPV [58]. Oral sex is the main transmission root in these diseases, and soft-circumscribed sessile nodular lesions and koilocytosis are some of their pathological and cellular manifestations [59]. The latent location of HPV in the mouth is usually in the gingival pocket because it is the only place where basal cells are in direct contact with the environment [60]. In about one-fourth of patients with periodontal disease in a clinical survey, the gingival samples have been associated with HPV [60]. Additionally, alveolar bone loss has been related to tumor-positive HPV [60,61].

### 3.3. Risk Factors

Risk factors for HPV infection include unsafe sexual behavior, smoking, periodontitis, and inflammation. These factors affect HPV infection by altering the oral environment. Cigarettes stimulate and suppress immune mechanisms in the mouth through the chemicals in tobacco. In women with oral HPV, the prevalence of HPV-infected cervixes is also higher, which may be a factor in increasing the risk of oral HPV [62].

### 3.4. Diagnosis

There are several diagnostic methods for HPV. Immunohistochemical analysis-specific antibodies (e.g., p16INK4A and p16 IHC) and HPV mRNA/DNA-detecting PCR are the sensitive and cost-effective diagnostic methods for HNSCC tumor specimens. However, studies have shown that mRNA tests are the best approach for confirming the diagnosis [63]. Serological biomarker tests cannot be used for detecting HPV infection in the oral cavity. Their examination in oral fluids is useful for identifying and examining the incidence and course of the disease as they are low-cost, non-invasive, and local-specific [64].

### 3.5. Treatment

Syrjänen discusses that HPV particles only get inactivated at temperatures 75–80 °C [62]. Preventive approaches such as vaccination and routine screening of HPV antibodies in the saliva are among the most effective ways to prevent HPV-associated head and neck diseases. Cold therapy, electrosurgery, surgical resection, laser therapy, and trichloroacetic acid are the usual treatments for papillomas/condyloma, verocroas, and FEH occurred by HPV [52]. The therapeutic developments in treating HPV infection are summarized in Table 1.

## 4. *Candida albicans*

More than 200 species of the genus Candida are usually non-pathogenic in humans. However, in immunocompromised individuals, *Candida* is the most frequent cause of oral mucosal infections, commonly due to antibiotics and the consequent change in the bacterial microbiota. In addition, suppressing the local or systemic immune system prepares the environment for infection. This group’s most common pathogen species is *C. albicans*, which accounts for more than 90% of oral lesions [65]. Morphological control between yeast and hyphae by *C. albicans* is involved in its pathogenesis. *C. albicans* co-adhesion with oral bacteria such as *S. mutans* helps it to colonize, persist, and grow by receiving a carbon supply. Vice versa, *Candida* reduces the bacteria’s oxygen stress, promotes better bacteria adhesion, and prepares stimulatory growth factors [66,67].

### 4.1. Epidemiology

The general prevalence of *C. albicans* is 20–75%, with 95% incidence in HIV patients, 90% in patients undergoing chemotherapy and acute leukemia, 88–65% in patients at long-term caring centers, 50–65% in people with detachable dentures, 45–30% in healthy adults, 65–45% in healthy children, and 45% in infants. In immunocompromised individuals, severe systemic exposure to *Candida* species increases mortality [68]. Oropharyngeal *Candida* is an opportunistic and recurrent fungal infection in more than 90% of patients with HIV [69]. The mortality rate of *Candida* bloodstream infection (candidemias) is more than 30% [68].

### 4.2. Clinical Presentation

Pseudomembranous, erythema, hyperplasticity, mucosal irritation, and edema are the symptoms of primary oral candidiasis [70,71]. In more severe cases, hemodynamic instability, positive blood cultures, fever, shock, and tachycardia may occur [70]. *Pseudomembranous Candidiasis* (thrush) is found on the white plaques, buccal mucosa, oropharynx, and junction of the hard and soft palate and is the most common form of candidiasis. Although some patients may experience a sour taste, burning sensation, and bleeding in the affected areas, most patients are asymptomatic [71]. Candidiasis may be hyperplastic or atrophic and occurs in either chronic or acute forms [70,72]. Different types of candidiasis can be asymptomatic or ulcerous [70]. Hyperplastic candidiasis resembles leukoplakia and may involve the labial commissures and become malignant [72]. Its chronic form is challenging to diagnose since the hyphae may hide in any rough surface in the oral cavity, such as papilloma, epithelial dysplasia, and squamous cell carcinoma [72]. Acute atrophic candidiasis is usually iatrogenic (such as antibiotic consumption) and especially common in HIV patients, whereas chronic atrophic candidiasis is iatrogenic mainly due to orthodontic retainers or dentures that do not adequately fit in place [70]. Chronic atrophic candidiasis and inflammatory papillary hyperplasia [73] may also accompany due to iatrogenic candidiasis because of taking biopsies from median rhomboid glossitis, smoking, and inhaled steroids, for instance [70]. Symptoms of esophageal candidiasis include ulceration, obstruction, narrowing of the esophagus, chest pain, nausea, and vomiting [70]. *C. albicans*, *S. aureus*, and β-hemolytic *Streptococcus* species are among the microorganisms present in angular cheilitis. The anterior region of the nostrils to the corners of the mouth is the source of staphylococci. Iron deficiency anemia, and vitamin B12 deficiency are among the etiological factors [74].

### 4.3. Risk Factors

IgA, mucins, and antifungal agents (e.g., histatin 5 and calprotectin) protect the oral environment from *Candida* infection. However, factors such as age, organ transplants, HIV, chemotherapy, poor oral hygiene, smoking, inhaled steroids, and radiotherapy increases its risk [75]. Then, in all patients with daily dental care, treatment of xerostomia, control of diabetes, restriction of corticosteroids and topical or systemic antibiotics, and elimination of underlying predisposing factors are very important [76]. The oral amount of *Candida* in HIV patients relates to their salivary flow [77]. The overgrowth of *Candida* species has no significant association with oral bacterial dysfunction [75].

### 4.4. Diagnosis

Candidiasis suspicion is based on examining mucosal changes, stained smears with Schiff’s reagent or KOH, and histopathological biopsies, searching for the hyphae or epithelial parakeratosis with polymorphonuclear leukocytes [78]. Transcribed internal distance sequencing [40] can be used to identify emerging candidate species and the historical course of candidiasis. For the rapid diagnosis of invasive candidiasis, serological biomarkers, including antibodies against mycelium, β-d-glucan (BDG), mannan antigen (Mannan-Ag), and mannan antibodies, are evaluated [79]. Endoscopy is also necessary for esophageal candidiasis suspicion patients [70].

### 4.5. Treatment

Polyenes and azoles are two types of antifungal drugs [80,81]. Polyenes (with conjugated double bonds) bind to sterols (mainly ergosterols) and change the cell membrane transfer temperature. Then, the leakage of monovalent ions (K^+^, Na^+^, H^+^, and Cl2) and small organic molecules lead to cell death [80]. On the other hand, azole stores α-methylase enosterol to prevent the lanosterol to ergosterol conversion. These topical drugs have no pharmacological systemic side effects since they have no systemic absorption. In cases where topical drugs do not respond, systemic drugs are used [81]. Studies have shown that probiotics may affect the toxicity of *C. albicans*. For example, *Lactobacillus* spp. and *S. salivarius* prevent the differentiation of *C. albicans* into hyphae or biofilm structure and decrease the salivary yeast level. Probiotics also increase the level of anti-*Candida* IgA and, consequently, the yeast level [82]. Recent developments for *candida* infection are shown in Table 2.

## 5. *Aspergillus*

*Aspergillosis* genus is the second most common opportunistic fungal infection in humans. *Aspergillus fumigatus* is an air-borne fungal pathogen causing many diseases [108,109]. This pathogen has a saprotrophic mycelial with an efficient spread through asexual spores and a life mostly on decaying organic matter secreting a wide range of enzymes (e.g., amylases, xylanases, pectinases, and elastase) [108]. However, some studies have evidence of heterothallism and a periodical sexual mating procedure [110]. All isolates of *A. fumigatus* are highly hydrophobic and fast-growing, but their virulence can vary based on indefinite factors such as the growth rate of isolates [109].

The main virulence factors of *A. fumigatus* are its cell wall containing polysaccharides (90%) and proteins and the glutotoxin from the epipolythiodioxopiperazines family [108]. Through the pathogenesis of Aspergilloma (noninvasive chronic pulmonary Aspergillosis), *A. fumigatus* hyphae form a biofilm in the extracellular matrix (ECM) with a different cell wall composition and structure [110].

### 5.1. Epidemiology

The epidemiological studies report that the prevalence of Aspergillus varies in a geographical-, regional-, seasonal-, and occupational-dependent manner [111]. *Aspergillus* spp. has been isolated from the respiratory tract of asymptomatic patients, and its DNA has been found in 37% of healthy individuals [112]. Its prevalence has been 30% in patients with chronic obstructive pulmonary disease (COPD), and 53% of patients with *Aspergillus* positive culture were conflicted with invasive Aspergillosis (IA) [112]. Distal–lateral subungual onychomycosis (DLSO) is an onychomycosis condition due to *Aspergillus* spp., with a prevalence of up to 35% on average and 71% in the diabetic population. It also accounts for 18–50% of all nail diseases and 30% of fungal skin infections [111].

### 5.2. Clinical Presentation

*Aspergillus* colonization function damages the epithelial cells and upregulation of ECM proteins by disrupting the expression of the ZNF77 transcription factor in bronchial epithelium and causing conidial adhesion. The immune system-survived and metabolically activated conidia grow, germinate, form hyphae, spread by attacking blood vessels, and invade the lung tissue [113]. Aspergillosis is divided into three categories: invasive (nonfulminant and fulminant), noninvasive, and noninvasive destructive. The nonfulminant invasive types are slowly progressive, and the fulminant invasive types are very aggressive. The non-invasive type can be locally destructive but has no tissue invasion and includes Aspergilloma, fungal ball, and Mycetoma [78,114]. Headache, fever, nasal congestion, swelling of the face, purulent or bloody nasal discharge, nasal pain, and epitaxy are the clinical symptoms of *A. rhinosinusitis*. This diagnosis should be considered in people with regular sinusitis or who are resistant to antibiotics. Oral lesions associated with Aspergillosis and other systemic mycoses can be considered dispersed diseases of the lungs. Irregular oral lesions may indicate the spread of an adjacent structure, such as the maxillary sinus, or a significant infection of the oral mucosa [115]. In the first stage of *Aspergillosis*, marginal growth areas appear to contain degraded epithelium and infiltrate fungal hyphae in the connective tissue. In the next stage, the previous lesions change to necrotic gray lesions and spread by attachment to the gums by ulceration and pseudomembrane. Invasion of the arteries is found at the base of these wounds. In the last stage, progressive damage to the alveolar bone and muscles is characterized by histopathological evidence of the penetration of fungal hyphae around the face [116]. Poor outcomes were associated with cases of older age, bone marrow transplantation, high sequential organ failure assessment (SOFA) score, and mechanical ventilation [112].

### 5.3. Diagnosis

To diagnose Aspergilloma, chest radiographs are still a suitable imaging technique that shows a round solid body enclosed in a radiolucent crescent in the upper part of the lung (bilateral and multiple). Thin-section chest computed tomography (MDCT), multiple incision (MSCT), spiral computed tomography (CT), and high-resolution CT at the optimal dose are suitable methods for patients at risk of IA. In the early stages of IA analysis, CT lung angiography can show vascular occlusion at the level of a suspected fungal lesion [117]. Additionally, microscopic examination and culture should be performed in patients at risk for IA. Culture is not a specific and appropriate diagnostic marker, while sputum culture is positive for *Aspergillus* in half of the patients with Aspergilloma. In the microscopic examination, it is essential to prove tissue attack by hyphae. The sensitivity of microscopic examination for IA is about 50% [118]. Evaluation of anti-*Aspergillus* antibodies by immunoblots and enzyme-linked immunosorbent assay (ELISA) methods is available as a robust and inexpensive method which is the reference method for serological diagnosis of chronic pulmonary Aspergillosis (CPA) [119].

### 5.4. Treatment

The standard doses of anti-fungi drugs recommended for treating IA may not be safe or effective for all patients. Then, high doses of drugs are commonly required in severe infectious diseases, treatment of difficult places, and infections caused by *Aspergillus* spp. with increased MIC. Patients with hematological malignancy at risk for IA are also managed by receiving initial prophylaxis or controlling biomarkers without receiving prophylaxis [120]. Oral-delivered Raziol treats CPA. All treatment instructions for the invasive Aspergillosis include using azoles, Amphotericin B (AmB), or echinocandin at appropriate doses with therapeutic evidence. However, in some regions, high rates of triazole resistance have been observed in *A. fumigatus*. In patients without previous exposure to antifungal therapies, a worrying resistance rate of 6% has been reported [108]. Recent developments in treating Aspergillosis are presented in Table 2.

## 6. *Actinomyces*

The genus *Actinomyces* spp. belongs to the typical human flora that can be found in the oropharynx, gastrointestinal tract, and urinary tract. It is one of the leading oral bacteria usually identifiable in healthy dental mucosa, dental plaque biofilm, periodontal lesions, and root rot. Actinomycosis resembles malignancy, tuberculosis, or nocardiosis in terms of its continuous and gradual spread [121]. The most common species in clinical conflicts is *Actinomyces israelii*. Actinomycosis infections can be associated with companion microbes inhibiting the host defense or reducing oxygen stress. The onset of Actinomycosis is likely to be multifactorial, and its association with mucosal cleavage is unpredictable. For example, minor trauma to the mucosa caused Actinomycosis in one patient, while no significant actinomycosis was observed in the large ruptures of the mouth [122].

### 6.1. Epidemiology

In 60% of all patients, Lmpy jaw syndrome (or action) is associated with odontogenic infection. The most common clinical form is cervicofacial Actinomycosis, and the most prevalent species are *A. israeli* and *A. gerencseriae*, *A. naeslundii*, *A. viscosus*, *P. propionicum*, *A. gerencseriae*, and *A. odontolyticus* [122]. Actinomycosis is more common in rural areas, men, and middle-aged people (50–30 years). The mortality rate is between 0 and 28% [123].

### 6.2. Risk Factors

Actinomycosis is associated with dental procedures. Diabetic patients are more likely to get infections because they have structural changes in their tissues and have impaired wound healing. Tumors, surgery, and radiation may also cause local tissue damage to spread into this infection [124].

### 6.3. Clinical Presentation

Complete vascularization of mucosal tissues and their replacement by weakly irritated tissue in actinomycetes supports its growth and provides adequate oxygen pressure. In necrotic foci, filamentous “sulfur” granules spread as a “sunburst radiation”. The ends of these granules can form extensions or rosettes due to the adhesion of neutrophils [123]. Cervicofacial clinical symptoms, which may last from 4 days to 1 year before diagnosis, include irregularly painful soft-tissue swelling of the submandibular or perimandibular area and emptying of the sinus ducts with sulfur granules, chewing problems, and recurrent and chronic infection [123]. In about 10% of patients, the bone is involved. Chronic infection can lead to osteomyelitis of the jawbone. Osteomyelitis due to cervicofacial Actinomycosis can spread to the lungs, gastrointestinal tract, tongue, sinuses, middle ear, larynx, ciliary tract, and thyroid gland [125].

### 6.4. Diagnosis

The best diagnoses are histological examination and bacterial culture of abscesses or suspected tissue. Staining sulfur granules with hematoxylin–eosin turns them into round basophil masses containing eosinophilic terminal clubs. Prescribing antimicrobial drugs leading to false negative culture results may cause anemia, mild leucocytosis, increased erythrocyte sedimentation rate, and increased C-reactive rutin value. Increased alkaline phosphatase concentrations may be seen in patients with hepatic Actinomycosis [126]. The blood test is a nonspecific diagnostic method for this disease. Imaging features are nonspecific and non-diagnostic in the early stages and may even be related to other inflammatory processes or neoplasms. Although cross-sectional imaging with CT or magnetic resonance imaging (MRI) does not provide accurate or diagnostic information, it can provide accurate anatomical information for sampling. Regional lymphadenopathy is rare in these patients. In later stages, infiltration of the surrounding tissues may be observed that is not diagnostic again. An aspiration biopsy should be performed in oscillating abscesses to examine the pus in the sulfur granules [127].

### 6.5. Treatment

Depending on the infection course, the course of antibiotics determines the clinical manifestations and response in Actinomycosis. The treatment is experimental because no similar success has been achieved with any antibiotic. The use of high doses of intravenous antibiotics for 2–6 weeks or 6–12 months orally is the primary treatment [128]. Acute lesions are often treated with tooth extractions, abscess drainage, and antibiotics for 2–3 weeks (penicillin). The penetration of antibiotics into the lesion may be delayed by weak vessels and solid capsules. Surgical interventions such as bone necrosis removal are performed for subacute or chronic voluminous lesions [129]. The recent treatments for Actinomycosis are summarized in Table 3.

## 7. *Streptococcus mutans*

*S. mutans* lives in the mouth, specifically on dental plaque. Its importance is for involvement in the etiology of dental caries and its possible association with subacute infective endocarditis. Studies have shown that *S. mutans* is a major cause of tooth decay because of its ability to make large amounts of organic acids and activity at low pH compared to other species [154,155,156]. Through pathogenesis, *S. mutans* develop a biofilm starting by attachment of the initial pioneer species followed by colonization, co-adhesion, and co-aggregation of other species. Then, the bacteria produce extracellular polysaccharides, separate from the biofilm surface, and spread in the oral cavity environment [157]. *S. mutans* produces a sticky glucan by the action of glucosyltransferases (GTF) on sucrose that helps bacteria tight binding to the tooth surface. This binding allows bacteria to withstand rapid and frequent environmental fluctuations such as nutrient access, aerobic to anaerobic transfer, and pH changes. *S. mutans* also produces other virulence factors, including glucan-binding (Gbp) proteins and antigenic cell surface protein (PAc). PAc is in contact with salivary glands and plays an essential role in bacterial adhesion to tooth surfaces [158].

### 7.1. Epidemiology

Permanent tooth decay did not change according to age standards between 1990 and 2010 and peaked between 25 and 70, respectively. No significant differences were observed in terms of gender. Untreated tooth decay is the most common health condition worldwide. Studies have shown that the prevalence of early childhood caries is worrying in various regions worldwide [159].

### 7.2. Clinical Presentation

Tooth decay, the leading cause of tooth loss, is a multifactorial, infectious, and transmissible disease [160]. According to plaque-specific plaque (SPH) hypotheses, certain Gram-positive acidogenic and aciduric bacteria, including *S. mutans* and *S. obrinus* are typical infective dental plaques causing tooth decay as a biofilm-mediated disease in humans [161]. Environmental conditions such as regular daily sugar intake or salivary dysfunction increase the aciduric/acidogenic oral microbiome. As the lesions spread, the physiological balance between the tooth mineral and the biofilm fluid is disturbed, moving toward demineralization [162].

### 7.3. Diagnosis

Caries is diagnosed by visual and tactile dental examination. Alternative methods, including illumination-based methods such as optical coherence tomography [71], near-infrared [116], and fiber-optic technology, are also available [163]. In addition, the quantitative fluorescence light (QLF) devices, categorized by red, blue, and green labels based on the various wavelengths they generate, can be used in the early stages of caries [164]. Another method is an electronic caries monitor (ECM) that measures the bulk resistance of dental tissue. Material properties such as porosity, contact area, tissue thickness, enamel hydration, and ionic content of tooth fluids determine its electrical conductivity. Visual and radiographic assessments are used alongside other methods such as Diagnodent, ECM, or QLF. However, it is unclear whether such methods can be used as a standard tool in diagnosing tooth decay [165].

### 7.4. Treatment

First, biofilm management should be considered before tissue removal [166]. Patients are advised to consume less fermentable carbohydrates to correct the environmental pressures responsible for plaque biofilm dysbiosis [167]. Recent therapeutic advances are shown in Table 3.

## 8. *Streptococcus sanguinis*

*S. sanguinis* is a member of the Streptococcus family and a Gram-positive and facultative anaerobe. Similar to other streptococci, *S. sanguinis* divides along a single axis. According to reports, *S.sanguinis* is nonmotile. *S.sanguinis* use several carbohydrate sources to sustain itself. During the eruption of the first teeth of toddlers, *S. sanguinis* colonizes the oral cavity. Streptococcus species, however, have been reported to form biofilm during the first four to eight hours following biofilm formation. Their colonization of enamel is pioneering, particularly that of the mitis. As a result of refraining from using a toothbrush for some time, *Streptococcus* spp. colonizes the enamel more quickly, which could contribute to its cariogenic nature. *S. sanguinis* may colonize a mature biofilm at different rates, resulting in different cariogenic traits [168].

### 8.1. Epidemiology

Commensal bacteria such as *S. sanguinis* are common in the mouth. In human saliva, it can be found on tooth surfaces and the surface of the oral mucosa. In both supragingival and subgingival plaque, *S. sanguinis* thrives as an anaerobic species. Despite similar plaque mass, *S. sanguinis* biomass can differ substantially at different tooth locations. Teeth with lower incisors/canines have a high proportion of it, but upper molars with low proportions. Various dental implant surfaces have also been shown to form biofilms due to *S. sanguinis* [169].

### 8.2. Mechanism of Action

In general, *S. sanguinis* and *S. gordonii* are less acid-tolerant than *S. mutans*, but they contain arginine deiminases, which produce ammonia and provide ATP when exposed to acidic conditions. This system improves the survival and persistence of these organisms. Researchers have found that bacterial uptake and catabolism of specific carbohydrates can affect H_2_O_2_ and AD production by these commensals [170].

### 8.3. Clinical Importance

Dental caries has previously been shown to be prevented by *S. sanguinis*. For many years, *S. mutans* and humans have been antagonistic. There is a significant correlation between early *S. sanguinis* colonization and later *S. mutans* colonization. *S. sanguinis* levels decrease after *S. mutans* colonization. The inhibiting ability of *S. sanguinis* strains against Prevotella intermedia BS6 was found in at least one-third of the strains tested. As a result of hydrogen peroxide production by *S. sanguinis*, *A. actinomycetemcomitans* cannot grow in vitro [171]. Microbial and HSP epitope mimicry and microbial and HSP colonization of the oral mucosa have been proposed to elicit immunity and autoreactive T-cell clones. HSP stimulates adaptive and natural immune responses. Several autoimmune diseases, such as Behcet’s disease, have been linked to oral bacteria, including *S. sanguinis* [172].

### 8.4. Diagnosis

It has been primarily physiological and biochemical characteristics used to identify *S. sanguinis* in the past. Nevertheless, phenotypic identification methods and investigators differed in reliability and reproducibility. Previously, genotypic and phenotypic methods did not accurately identify clinical *S. sanguinis* isolates. To identify *S. sanguinis* and other oral bacteria, other methods, such as PCR with nucleic acid probes, are being investigated. [173].

## 9. Conclusions

Today, protecting and adequately repairing the tooth structure is the most critical approach to caries management. Successful clinical management of dental caries requires a good biological understanding of the disease process and the associated microbiome. Current approaches are based on the International Caries Consensus Collaboration (ICCC) recommendations, which state that tooth decay is a biofilm disease. In this regard, the literature states that biofilm management should be focused on before tissue removal to prevent the formation of new lesions and control the previous lesions [166]. Then, an understanding of microbiology, anatomy, pharmacology, wound healing, and oral surgery is required to manage acute dental infections. Today, the natural balance of the oral microbiome can be altered by lifestyle. The clinical goal in these patients is to establish this balance.

Despite treatment according to protocols, severe infections can be life-threatening. Therefore, using the idea of oral microbiome balance is essential for patients and healthcare professionals. The first approach is to maintain health rather than disease management. In preventing and diagnosing oral infections, healthcare providers can be very effective with activities such as controlling infections according to new protocols, educating patients, raising awareness about proper nutrition and uncontrolled sexual relationships, and motivating regular checkups. Early detection of infection and pathogens helps reduce oral or general infection. Identifying all aspects that cause antimicrobial resistance, immune system volatility profiles, potential mutations, and genotype differences should be examined in managing these infections. Timely management prevents complications in the patient and prevents the spread of the disease to others. In addition to their skills in treating these infections, clinicians need evidence-based knowledge about the pros and cons of any treatment.

## 10. Future Directions

It is suggested to study the mechanisms controlling the fate and function of infected tissues. Meta-analysis studies and robust trials with long follow-up periods should be performed, considering individual differences and other clinical limitations. Using the results of these studies, develop new treatments and approaches for tooth decay or damage to teeth and orofacial tissues. Several strategies have been used to prevent and manage oral infections in some not-yet-published current clinical trials. Genetics is also suggested as an important underlying factor that can affect the spread of infection and the immune response. Today, with the advancement of modern imaging systems and rigorous laboratory experiments, researchers can examine their treatment methods and disagreements in a shorter time.

## Figures and Tables

**Table 1:** Recent developments in treating orofacial viral infections.

Species	Samples	Intervention	Outcomes	Ref. Year
HPV	Patients with progressed oropharyngeal cancer	Accelerated fractionation radiotherapy and standard-fractionation radiotherapy	Over 6o percent of patients were HPV-positive and showed better overall survival in 3 years compared to HPV-negative tumors.	[31] 2010
HSV-2	Patients with acute primary or recurrent HSV-2	1 g valacyclovir 3 times daily for 1 week, followed by 0.5 g valaciclovir twice daily for 1 year.	The following-up prescription showed insufficiency in prohibiting recurrent meningitis.	[16] 2012
HPV	Patients under cervical surgery	Three doses of quadrivalent HPV vaccine or placebo at days 1, 60, and 180	Quadrivalent HPV vaccination after surgical treatment significantly reduced recurrent HPV-related diseases.	[32] 2012
HSV-1 and 2	Patients with HIV-1 and HSV-2	Valacyclovir 1000 mg or acyclovir 400 mg twice a week for ~3 months	High-dose valacyclovir was more successful in reducing the plasma HIV-1 RNA levels compared to the standard dose.	[33] 2013
HSV-2	Patients with HIV and HSV-2 in co-infection	Patients randomly received valacyclovir or placebo (N = 35)	The CD4+ T-lymphocyte count or HIV viral load did not change, but asymptomatic HSV-2 shedding reduced slightly.	[34] 2014
HSV-1 or HSV-2	RCTs	Effectiveness of oral antiviral drugs (acyclovir, famciclovir, and valacyclovir)	Researchers found there was a significantly lower number of patients with at least one genital herpes recurrence when acyclovir, valacyclovir, or famciclovir was used to treat patients with at least four recurrences per year as compared with placebo in patients with at least four recurrences.	[35] 2014
HPV	Patients examined for gingivitis	Three medicinal mushrooms: *Laetiporus sulphureus*, *Ganoderma lucidum*, and *Trametes Versicolor*	*Laetiporus sulphureus* exerted 5% oral HPV clearance, while *Trametes Versicolor* plus *Ganoderma lucidum* showed a clearance of 88%.	[36] 2014
HPV-16 and HPV-18	Patients from 36 gynecology practices in seven countries	6 mg VGX-3100 or placebo	This vaccine is the first to show effectiveness against CIN2/3 associated with HPV-16 and HPV-18 and provides a new treatment outlook.	[37] 2015
HSV-2	Healthy adults with recurrent genital HSV-2	100 mg oral pritelivir with 500 mg valaciclovir once a day	People with frequently recurrent genital HSV-2 using pritelivir experienced a lower percentage of HSV+ swabs.	[38] 2016
HPV	Patients with high-risk cervical HPV infection	An anti-HPV biological dressing (JB01-BD)	JB01-BD could effectively decrease the viral load.	[39] 2016
HSV-1 and 2	Patients with post-herpetic neuralgia	200 mg or 400 mg valnivudine hydrochloride (FV-100) once daily, or 1000 mg valacyclovir three times daily	Treatment with FV-100 decreased the neuralgia in a dose-dependent manner better than valacyclovir.	[40] 2017
HSV-2	Adults with symptomatic HSV-2	30 or 60 µg antigen against glycoprotein D2 and viral transcription factor ICP4.2	The GEN-003 vaccine combinations with higher amounts of antigen and adjuvant showed more efficacy.	[41] 2018
HSV-2	Patients with recurrent genital HSV-2	A vaginal capsule of multistrain *Lactobacillus brevis* or oral acyclovir	Probiotic therapy with multi-strain *L. brevis* was a promising low-cost treatment for recurrent genital herpes simplex virus infection compared with acyclovir.	[42] 2018
HPV	Patients with multiple common warts	Intramuscular and intralesional bivalent HPV vaccine	Both HPV vaccination roots showed potential for treating warts.	[43] 2019
HSV-1	Patients with herpetic stromal keratitis	Topical cyclosporine-A 2% eye drop with prednisolone acetate 1% eye drop	Both treatments could similarly improve the cornea’s optical density to a significant extent.	[44] 2019
HPV	Patients with genital condylomatosis	Dry extracts of *Echinacea purpurea* and *Elaeagnus angustifolia* (HPVADL18^®^)	HPVADL18^®^ was suggested as a potential adjuvant therapy for reducing recurrent lesions after treating genital condylomatosis.	[45] 2019
HPV	Patients with high-risk HPV	A proprietary combination of antiviral biologics (REBACIN^®^)	The antiviral agent could significantly repress the expression of E7 and E6 oncogenes in HPV and clear persistent HPV infections.	[46] 2019
HSV-1 and 2	HIV-1-positive adults	500 mg valaciclovir twice daily	Valaciclovir modestly lowered the HIV viral load but did not slow the CD4 count decline.	[47] 2019
HPV	Patients with recurrent respiratory papillomatosis	10 mg/kg avelumab every 2 weeks for three doses	Avelumab treatment led to fewer surgical interventions and reduced HPV viral load.	[48] 2019
HSV-1	Schizophrenic patients with or without HSV-1	1.5 g valacyclovir or placebo for 16 weeks	Valacyclovir showed no effect on the viral infection. HSV-1 infection co-occurred with a more severe form of schizophrenia.	[49] 2019
HSV-1 and 2	Patients receiving mechanical ventilation for over 4 days	Intravenous acyclovir 5 mg/kg	Acyclovir did not decrease the duration of mechanical ventilation and did not increase the number of ventilator-free days in patients with HSV oropharyngeal reactivation.	[50] 2019
HPV	Patients with anogenital warts	Podophyllotoxin cream 0.15% or imiquimod cream 5% with vaccination	Imiquimod and podophyllotoxin creams could similarly clear the wart, but the vaccine benefit was not observed.	[51] 2020

**Table 2:** Recent developments in treating fungal infections.

Species	Sample	Intervention	Outcomes	Ref. Year
*C. albicans*	Children under treatment with a removable maxillary appliance	NitrAdine tablets	The treatment had no significant effect on the salivary *Candida* load.	[83] 2011
*Aspergillus*	Patients with allergic bronchopulmonary Aspergillosis	Omalizumab	The treatment was successful in preventing the exacerbation of the infection.	[84] 2014
*C. albicans*	Adults living in nursing homes	Probiotics, including *Lactobacillus reuteri* (strains DSM 17938 and ATCC PTA 5289)	The probiotics significantly reduced the oral *Candida* counts.	[85] 2015
*C. albicans*	In vitro study	Pomegranate peel extract (PomeGr)	The PomeGr treatment altered biofilm formation, fungal growth, and AI release. Moreover, fungal cells substantially reduced PomeGr’s phenolic content	[86] 2022
*Aspergillus*	Patients with non-dermatophyte mold onychomycosis	Traconazole or terbinafine	Both treatment efficacy was non-statistically significant (clinical cure of 54–65%).	[87] 2016
*Aspergillus*	Patients with suspected invasive mold infection	Intravenous injection of isavuconazonium sulfate or voriconazole followed by further oral administration	Isavuconazole efficacy was not worse than voriconazole. It was well tolerated with fewer adverse events.	[88] 2016
*C. albicans*	Patients wearing dentures	Triphala churna and chlorhexidine gluconate	Triphala showed a more antifungal effect than conventional chlorhexidine.	[89] 2017
*C. albicans*	Patients with denture stomatitis	Low-molecular-weight chitosan and nystatin	Chitosan solution showed a significant antifungal effect.	[90] 2017
*C. albicans*	Patients wearing dentures	Two probiotics (*Lactobacillus acidophilus* or *Lactobacillus rhamnosus*) enriched into cheese	The enriched cheese with probiotics reduced oral *Candida* colonization.	[91] 2017
*Candida* and *Aspergillus*	Patients with candidemia and invasive candidiasis	A member of echinocandins (CD101 IV)	The dosing of CD101 IV was safe, minimally accumulative, plasma-persistent, and well-tolerated with negligible renal excretion.	[92] 2017
*C. albicans*	Patients with symptomatic oral lichen planus	Probiotics, including *Lactobacilli reuteri*	The probiotic used did not affect the *Candida* load.	[93] 2018
*Aspergillus* and *C. albicans*	Patients with otomycosis	Topical betadine and clotrimazole	The agents showed similar antifungal potential for treating otomycosis.	[94] 2018
*Aspergillus*	Patients in the acute stage of allergic bronchopulmonary Aspergillosis	Oral administration of itraconazole or prednisolone	Prednisolone induced a better immunologic response but more side effects.	[95] 2018
*C. albicans*	Patients with denture stomatitis	Photodynamic inactivation using a diode laser and methylene blue	The inactivation operation reduced the fungal and inflammation levels.	[96] 2018
*C. albicans*	Patients with candidemia or invasive candidiasis	Intravenous and oral isavuconazole comparedto caspofungin and voriconazole	Isavuconazole showed a lower minimal inhibitory concentration than caspofungin.	[97] 2019
*C. albicans*	In vitro study	Tissue conditioner modified by chitosan or chitosan-oligosaccharide	Both formulations reduced the *C. albicans* density.	[98] 2019
*C. albicans*	Patients wearing complete dentures with stomatitis	Photodynamic therapy using indocyanine green was added to the routine antifungal therapy with nystatin mouthwash alone	The combined therapy helped to improve the denture stomatitis showing no adverse effects.	[99] 2019
*S. mutans*, *C. albicans*, *C. glabrata*, and *C. parapsilosis*	In vitro study	Propolis, saline, or alkaline peroxide solutions	The propolis solution had an antimicrobial effect against *S. mutans* and *C. albicans*, showing no immediate effect on denture biofilm.	[100] 2019
*C. albicans*	Patients wearing removable dentures	Chitosan-curcuminoid/PEG mouthwash compared to chlorhexidine	The composite alcohol-free mouthwash was a safe topical therapeutic for treating *candida*-associated denture stomatitis.	[101] 2019
*Aspergillus*	Patients with chronic pulmonary Aspergillosis	Intravenous followed by oral administration of itraconazole	The treatments with itraconazole were effective on chronic pulmonary Aspergillosis.	[102] 2019
*Aspergillus*	Patients with invasive Aspergillosis	Single and multiple ascending intravenous doses of an antifungal drug (VL-2397)	The dosing of VL-2397 was safe, non-accumulative, and tolerable in both healthy subjects and patients.	[103] 2019
*C. albicans*	Patients wearing dentures with stomatitis	Photodynamic inactivation by GaA1As diode laser in comparison with nystatin	Both treatments were equally effective in treating denture stomatitis.	[104] 2019
*C. albicans*	Patients wearing maxillary dentures	Dettol and Lifebuoy liquid soaps compared to sodium hypochlorite and phosphate-buffered saline solution as positive and negative controls	The liquid soaps efficiently reduced the fungal biofilm.	[105] 2020
*C. albicans*	Patients after head and neck radiotherapy	Probiotics, including *L. acidophilus*, *B. longum*, *L. rhamnosus*, and *S. boulardii*	The probiotic bacteria could effectively reduce the oral *Candida* load.	[106] 2020
*Aspergillus*	Patients with otomycosis	Clotrimazole cream and tolnaftate solution	Clotrimazole improved otitis better.	[107] 2020

**Table 3:** Recent developments in treating bacterial infections.

Species	Sample	Intervention	Outcomes	Ref. Year
*S. mutans*	Healthy subjects	Chlorhexidine and garlic extract mouthwash	Garlic extract inhibited *S. mutans* in both in vitro and in vivo studies.	[130] 2010
*A. gerencseriae*	Patients with aggressive periodontitis	Systemic azithromycin	Azithromycin could slightly reduce the subgingival periodontal pathogens	[131] 2012
*Actinomyces* spp.	Smoker and non-smoker subjects	Metronidazole and Amoxicillin	The non-smokers showed the lowest proportions of the orange complex and a meaningful increase in the proportions of *Actinomyces* species.	[132] 2013
*S. mutans*	Children with decayed, missing, or filled teeth	Propolis and xylitol chewing gums	Both gums reduced the bacterial saliva load suggesting them an anti-cariogenic agent.	[133] 2014
*Lactobacillus* spp. *Actinomyces naeslundii* *S. mutans*	Healthy adult subjects	Beverages containing apple fiber and polyphenols from boysenberry	The apple-boysenberry beverage exerted the most reduction on the colonization and biofilm adhesion.	[134] 2017
*S. mutans*	Adolescent subjects with disabilities	Xylitol gum	The xylitol gum significantly reduced the caries rate.	[73] 2017
*S. mutans*	Young subjects	Mouthwashes containing chlorhexidine, xylitol, and chlorhexidine + xylitol	All mouthwashes effectively reduced plaque, gingivitis, and bacterial saliva level.	[135] 2017
*S. pseudopneumoniae* *A. odontolyticus*	Adults with bronchiectasis	Erythromycin	The intervention significantly decreased the oropharyngeal microbiota composition.	[136] 2018
*A. actinomycetemcomitans* *A. naeslundii* *A. viscosus* *E. faecalis* *E. coli* *L. casei* *S. oralis* *S. sanguinis* *C. albicans*	In vitro study	Several flavonoids	Among the eight tested flavonoids, morin was the most effective; however other four flavonoids, including luteolin, naringin, quercetin, and rutin, could also decrease bacterial and fungal growth.	[137] 2019
*A. israelii* *E. faecium* *F. nucleatum* *L. gasseri* *S. mutans* *V. parvula*	In vitro study	Curcumin, protoporphyrin IX, resazurin, riboflavin, and light irradiation	All tested agents decreased the oral bacterial growth.	[138] 2019
*E. faecalis* *S. gordonii* *A. naeslundii* *L. acidophilus*	In vitro study	Dimethylaminododecyl methacrylate (DMADDM) and EndoREZ	The two tested sealers showed similar cytotoxicity, apical sealing ability, and solubility; however, DMADDM showed different properties at a mass fraction of 5%.	[139] 2019
*S. oralis* *A. naeslundii* *V. parvula* *F. nucleatum* *P. gingivalis* *A. actinomycetemcomitans*	In vitro study	Red wine, dealcoholized red wine, polyphenols-rich extracts from wine, and polyphenols-rich extracts from grape seeds	Conventional and dealcoholized red wine reduced the bacteria load within the biofilm, especially about *P. gingivalis* and *A. actinomycetemcomitans*.	[140] 2019
*S. mutans* *A. naeslundii*	In vitro study	Surface pre-reacted glass-ionomer filler in a resin-based composite incorporated with 2-methacryloyloxyethyl phosphorylcholine	The polymer added in the filler composite inhibited bacterial attachment and biofilm growth.	[141] 2019
*A. naeslundii* *E. faecalis*	Extracted single-rooted human mandibular first premolars	Platelet-rich fibrin scaffold with or without a 3-antibiotic mixture containing minocycline, metronidazole, and ciprofloxacin	The combination of scaffold and antibiotic mixture showed the highest antibacterial activity.	[142] 2020
*S. mutans* *L. casei* *A. naeslundii*	Human mandibular third molars	Diode laser and 2% chlorhexidine gluconate solution	All three cariogenic bacteria were affected by a diode.	[143] 2020
*S. oralis* *A. naeslundii* *V. parvula* *F. nucleatum* *P. gingivalis* *A. actinomycetemcomitans*	In vitro study in a subgingival biofilm model	Doxycycline, zinc, and calcium doped polymeric nanostructured membrane that is non-resorbable	The nanostructured membrane significantly reduced the biofilm growth dynamics and bacterial load.	[144] 2020
*S. mutans* *S. gordonii* *S. sobrinus* *A. naeslundii* *F. nucleatum* *A. actinomycetemcomitans* *P. gingivalis* *E. faecalis*	In vitro study in planktonic culture	Fruit juices derived from blackcurrant, redcurrant, cranberry, and raspberry	Blackcurrant, redcurrant, and cranberry juices had the most suppressing effect on bacterial growth, respectively, while raspberry only significantly suppressed the growth of *P. gingivalis*.	[53] 2020
*S. mutans*	In vitro study on extracted teeth	A composite of nano-calcium fluoride and dimethylaminohexadecyl methacrylate	The composite showed promising fluoride release features and antibacterial functions.	[145] 2020
*E. faecalis* *S. mutans* *A. israelii*	In vitro study	Three licorice-derived polyphenols and cinnamon oil	The tested natural plant-derived compounds showed promising root canal disinfection properties.	[146] 2020
*S. mutans* *S. sobrinus* *A. viscosus* *L. acidophilus*	In vitro study	Caffeic acid phenethyl ester	The tested agent exerted high bactericidal and inhibitory activities against biofilms, cariogenic bacteria, and their virulence.	[147] 2020
*S. mutans* *A. naeslundii*	Human dentin blocks Periodontitis animal model	Surface pre-reacted glass-ionomer nanofillers	The tested nanofiller coating showed antibacterial effects on the tooth surfaces and improved the clinical parameters of periodontitis.	[148] 2021
*S. sanguinis*	In vitro study	The concentration of H_2_O_2_	*It has been shown that *S. sanguinis* evades neutrophil killing* in vitro *and counteracts innate immunity by the action of SpxB in collected blood*	[149] 2017
*S. salivarius*	Induced oral mucositis by experimental radiation in mice	The mouse oral cavity was treated daily with *S. salivarius* K12	Cancer patients receiving radiotherapy may benefit from *S. salivarius* K12 as an adjuvant treatment.	[150] 2021
*S. salivarius*	Primary human gingival fibroblasts	Pathogen-induced fibroblasts were treated with *S. salivarius* M18K12, K12, and fractions of its supernatant and whole-cell lysate	Periodontal disease pathogens were prevented from activating the immune system by S.salivarius M18 and K12. After chewing gum was administered with *S. salivarius* K12, the salivary microbiome and immune system did not change.	[151] 2021
*S. sanguinis*	In vitro study	Measurement of SsaACB manganese transporter	In acidic conditions, SK36 mutants lacking SsaACB display reduced growth and manganese uptake. *S. sanguinis* may have a variety of manganese transporters due to the heterogeneity of its oral environment.	[152] 2022
*C. albicans*, *S. aureus* and *P. aeruginosa*	In vitro study	Pomegranate (PomeGr) and microRepair (MicroR)	There were similarities in the effects of MicroR and PomeGr; however, the effectiveness of the two, given separately or in combination, varied based on which microbial agent was being treated.	[153] 2022

## Data Availability

Not applicable.
